# Neurologic manifestations in an infant with COVID-19

**DOI:** 10.1212/WNL.0000000000009653

**Published:** 2020-06-16

**Authors:** Rachelle Dugue, Karla C. Cay-Martínez, Kiran T. Thakur, Joel A. Garcia, Lokendra V. Chauhan, Simon H. Williams, Thomas Briese, Komal Jain, Marc Foca, Danielle K. McBrian, Jennifer M. Bain, W. Ian Lipkin, Nischay Mishra

**Affiliations:** From the Department of Neurology (R.D., K.C.C.-M., K.T.T., D.K.M., J.M.B.), Columbia University Irving Medical Center; Center for Infection and Immunity (J.A.G., L.V.C., S.H.W., T.B., K.J., W.I.L., N.M.), Mailman School of Public Health, Columbia University; Department of Epidemiology (W.I.L., N.M.), Mailman School of Public Health, Columbia University; Department of Pediatric Infectious Disease (M.F.), Columbia University Irving Medical Center; and New York Presbyterian Hospital (R.D., K.C.C.-M., K.T.T., M.F., D.K.M., J.M.B.), Columbia University Medical Center, New York, NY.

Currently, there are over 1.9 million confirmed cases of coronavirus disease 2019 (COVID-19) globally with over 590,000 cases in the United States.^1^ The number of COVID-19–positive children in the United States is unknown. A report summarizing 72,314 COVID-19 cases from the Chinese Center for Disease Control and Prevention noted 416 COVID-19–positive children younger than 10.^2^ An observational study at Wuhan Children's Hospital noted 31 COVID-19–positive children younger than 1 year with the youngest confirmed case in a 1-day-old.^3^ Cases were largely characterized by upper respiratory tract infection or pneumonia, fever, cough, and pharyngeal erythema.^3^ Concomitant neurologic problems have been reported among COVID-19–positive adult patients.^4^

We report the case of a COVID-19–positive 6-week-old who presented with fever, cough, and 2 brief 10–15 seconds episodes of upward gaze and bilateral leg stiffening.

## Case

A 6-week-old term male infant presented for evaluation after 1 day of cough, fever, and brief episodes of sustained upward gaze associated with bilateral leg stiffening. The initial episode occurred after a diaper change. There was no shaking, breathing change, or pallor during this episode. There was no association with feeding. The infant had 2 siblings with cough and fever at the time of presentation, both diagnosed with streptococcal pharyngitis. On arrival to the emergency department, vital signs were notable for fever of 38.4°C and mild hypertension (114/57). Examination was notable for a mottled appearance and bilateral overlapping of the fourth and fifth toes. The anterior fontanel was soft and nonbulging, and neurologic examination was unremarkable. However, the patient had a witnessed episode of sustained upward gaze associated with bilateral leg stiffening and decreased responsiveness lasting 10 seconds with subsequent return to baseline and no hypoxia or vital signs change.

The patient was born at 39 weeks via uncomplicated normal spontaneous vaginal delivery, weighing 3.91 kg. Family history was notable for simple febrile seizures in a developmentally normal sibling. There was no family history of epilepsy.

Laboratory data were notable for leukopenia of 5.07 ×10^3^ white blood cells (wbcs)/µL (normal 8.14–14.99 × 10^3^) with a normal differential and elevated procalcitonin of 0.21 ng/mL (normal <0.08 ng/mL). Electrolytes were normal. Respiratory pathogen PCR panel was positive for rhinovirus/enterovirus. SARS-CoV-2 Real-Time Reverse Transcriptase (rRT)-PCR was positive. A chest radiograph was not performed. A lumbar puncture had an unremarkable CSF profile with one red blood cell and 2 wbcs/µL, glucose of 50 mg/dL, serum glucose of 84 mg/dL, and protein of 40 mg/dL. Meningitis/encephalitis PCR panel was negative. CSF culture showed no cells nor organisms. Standard CSF testing does not detect COVID-19.

The patient was connected to long-term EEG monitoring which showed an excess of temporal sharp transients for age and intermittent vertex delta slowing with normal sleep-wake cycling. MRI of the brain with and without contrast to rule out a corresponding structural lesion was normal. Given the abnormal EEG findings and unexplained clinical events, further CSF, nasopharyngeal swab, serum, plasma, and anal swab testing were performed using high throughput sequencing and quantitative rRT-PCR after obtaining parental consent (see supplementary e-Methods, doi.org/10.5061/dryad.v41ns1rsc).

rRT-PCR nasopharyngeal swab testing was positive for SARS-CoV-2 RNA (∼2 × 10^6^ copies/mL). Anal swab testing showed low levels of viral RNA (∼200 copies/mL). SARS-CoV-2 RNA was not detected in CSF, serum, nor plasma. High throughput sequencing showed SARS-CoV-2 RNA and rhinovirus C sequences in nasopharyngeal and anal swab samples. *Moraxella* and *Corynebacterium*, common nasal flora, were detected in the nasopharyngeal swab (see supplementary e-Results, doi.org/10.5061/dryad.v41ns1rsc).

The patient was discharged home 1 day after admission without further fever or events on follow-up 1 week later.

## Discussion

The acute events reported in this case were characterized by sustained upward gaze, dystonic bilateral leg extension, and altered responsiveness in the setting of COVID-19 and rhinovirus. Although there was a strong initial suspicion for seizures, no events were captured on subsequent EEG to confirm this.

Infections, specifically pertussis and respiratory syncytial virus (RSV), have been diagnosed in up to 18% of infants with acute events, previously termed apparent life-threatening events.^5^ Most febrile seizures occur in the setting of viral infections; most commonly adenovirus, followed by influenza, rhinovirus, and RSV with coronavirus OC43 are detected in children younger than 12 months old.^6^

Our patient had COVID-19 in addition to rhinovirus confirmed on high throughput sequencing. Interestingly, a study of coronavirus-positive infants in the first 6 months of life noted a 27% coinfection rate with rhinovirus.^7^ There are reports of other pediatric coinfections with COVID-19.^8^

Despite the reports that children generally seem to have mild infection, this case report highlights the possibility of rare but important neurologic manifestations of COVID-19 in children. In addition, recognition of common coinfections will be important in guiding ongoing clinical evaluation and management of COVID-19–positive children.
